# In Memoriam—A. M. Leslie, D. D. S.

**Published:** 1867-03

**Authors:** 


### IN MEMORIAM—A. M. LESLIE, D. D. S.

At a meeting of the Dentists of St. Louis, held in the Missouri Dental College Infirmary, on the first of December, 1866, Dr. H. J. McKellops was called to the Chair, and Dr. E. Hale, jr.. appointed Secretary.

The death of Dr. A. M. Leslie was announced by the Chair, and a committee of three appointed to prepare and submit suitable preamble and resolutions expressive of our feelings on this sad and mournful dispensation of Divine Providence.

The sudden and unexpected close of an active and eventful life, in a career of enterprise and usefulness, can not fail to arrest the attention of the most thoughtless, and shroud an appreciative circle of acquaintances in the deepest gloom. Such was signally the case when the startling intelligence was flashed over the telegraphic wires, that our beloved LESLIE had died of cholera in the city of Memphis, on the morning of the 30th ult., in the 50th year of his age. Away from all the ties and warm endearments of home, and wife, and children, who loved and honored him in no ordinary degree, his death is truly saddening to our hearts.

A. M. LESLIE was a native of Edinburgh, Scotland. He was one of the first students in the Ohio College of Dental Surgery, in which he graduated in 1847, and soon rose to an honorable position in the faculty. He practiced his profession in Cincinnati until failing health compelled him to abandon its onerous duties and heavy responsibilities, when in 1856, he came to our city and established the Mississippi Valley Dental Depot; and, although he then became a manufacturer and dealer, he lost none of his love for the profession or sympathy with its practitioners. Nor was he any less desirous to see it made more useful, honorable and dignified by properly educating and elevating its members. To this noble enterprise he devoted much of his time, talent and means. His active and vigorous mind, suavity of manners, iron will and indomitable energy enabled him to accomplish a great deal of good.

In the formation and conducting of Dental Societies (of which he had large experience) Dr. Leslie was unsurpassed.

In the organization and starting of the Missouri Dental College he was a tower of strength, and stood unrivaled. One of the last acts of his life was to influence a student to come from a distant State and enter its halls. Therefore,

*Resolved*, That in the death of A. M. LESLIE, D. D. S., the Dental profession has sustained a great loss, and the Missouri Dental College one of its main pillars.

*Resolved*, That in token of our sorrow for his death, affection for his memory, and high appreciation of his many virtues, and the bright Christian example of our departed friend and brother, we will place these proceedings on record in the books of the Missouri Dental College, and publish them in the Dental journals of the country.

*Resolved*, That we hereby tender our true and heartfelt sympathy to the bereaved and sorrowing family and relatives of our deceased brother.

*Resolved*, That these proceedings be published in the *Missouri Democrat*, and copies, in gilt letters, on mourning paper, be furnished to the family. All of which is respectfully submitted.

H. E. PEEBLES, D. D. S.
AARON BLAKE, D. D. S. } *Committee.*
DR. ALEX. DIENST.

